# Dissemination of Resistant *Escherichia coli* Among Wild Birds, Rodents, Flies, and Calves on Dairy Farms

**DOI:** 10.3389/fmicb.2022.838339

**Published:** 2022-04-01

**Authors:** Rachel A. Hickman, Viktoria Agarwal, Karin Sjöström, Ulf Emanuelson, Nils Fall, Susanna Sternberg-Lewerin, Josef D. Järhult

**Affiliations:** ^1^Department of Medical Sciences, Zoonosis Science Center, Uppsala University, Uppsala, Sweden; ^2^Department of Medical Biochemistry and Microbiology, Zoonosis Science Center, Uppsala University, Uppsala, Sweden; ^3^Institute of Environmental Engineering, Zürich, Switzerland; ^4^Department of Clinical Sciences, Swedish University of Agricultural Sciences, Uppsala, Sweden; ^5^Department of Biomedical Sciences and Veterinary Public Health, Swedish University of Agricultural Sciences, Uppsala, Sweden

**Keywords:** antibiotic resistance, calves, rodents, flies, cross-species transfer, birds, dairy farms, livestock

## Abstract

Antimicrobial resistance (AMR) in bacteria in the livestock is a growing problem, partly due to inappropriate use of antimicrobial drugs. Antimicrobial use (AMU) occurs in Swedish dairy farming but is restricted to the treatment of sick animals based on prescription by a veterinary practitioner. Despite these strict rules, calves shedding antimicrobial resistant *Enterobacteriaceae* have been recorded both in dairy farms and in slaughterhouses. Yet, not much is known how these bacteria disseminate into the local environment around dairy farms. In this study, we collected samples from four animal sources (fecal samples from calves, birds and rodents, and whole flies) and two environmental sources (cow manure drains and manure pits). From the samples, *Escherichia coli* was isolated and antimicrobial susceptibility testing performed. A subset of isolates was whole genome sequenced to evaluate relatedness between sources and genomic determinants such as antimicrobial resistance genes (ARGs) and the presence of plasmids were assessed. We detected both ARGs, mobile genetic elements and low rates of AMR. In particular, we observed four potential instances of bacterial clonal sharing in two different animal sources. This demonstrates resistant *E. coli* dissemination potential within the dairy farm, between calves and scavenger animals (rodents and flies). AMR dissemination and the zoonotic AMR risk is generally low in countries with low and restricted AMU. However, we show that interspecies dissemination does occur, and in countries that have little to no AMU restrictions this risk could be under-estimated.

## Introduction

Any use of antimicrobials causes a selective pressure that favors resistant bacteria (Olesen et al., 2020). Hence, the global use and misuse of antibiotic drugs makes antimicrobial resistance (AMR) a growing problem, which is frequently reported in scientific literature and media outlets. The lack of treatment options due to antimicrobial resistance is most frequently discussed in regard to human health. However, AMR is also a problem in animal health, particularly in regions where these drugs are used as growth promoters or supplements in animal feed (McEwen and Fedorka-Cray, 2002; McEwen and Collignon, 2018). Animal-related AMR contributes to the continuous AMR dissemination and transmission that can cause several potential risks to animals and humans, e.g., biosecurity risks along the food chain, including resistant bacteria in food products (Aarestrup et al., 2008; Carmo et al., 2018). Antimicrobial-resistant bacteria in food products from livestock usually originate from the normal microbiome of healthy animals or, where lack of control leads to slaughter of sick animals, pathogens that contaminate the meat or food products from these animals (Cho et al., 2018; Jans et al., 2018) or from other sources, e.g., fruits and vegetables where feces from animals, or human wastewater, have been used as fertilizer to cultivate these foods (Mesbah Zekar et al., 2017; Karlsson et al., 2021).

In the European Union, the antimicrobial use (AMU) in animals has been declining since 2011 (European Medicines Agency year, 2019). In Sweden, already low, the levels of AMU in Swedish animals and humans have also declined since 2011 (SVA, 2019). However, global AMU is still rising, one major driver is the increased global demand for animal protein that has been facilitated by the expansion of intensive farming (Tiseo et al., 2020). There is a large global demand for bovine dairy and meat products despite local fluctuations (Lhermie et al., 2018). Despite good animal management procedures, animals often at some point during their life contract an infection that requires treatment with antimicrobial drugs, i.e., antibiotics, anthelmintics, or antifungals. Within dairy farms the most common illness that requires antibiotic treatment is mastitis (Cheng and Han, 2020). In most countries, milk withdrawal periods apply to dairy cows that undergo treatment, where milk cannot be delivered for human consumption. Such milk is often fed to calves or discarded into drains or on manure heaps (Firth et al., 2021). This can influence the calves’ microbiome and promote fecal shedding of resistant bacteria or lead to antibiotic drug residues selecting for AMR in the local environment (Duse et al., 2015).

From an international perspective, the Swedish dairy industry is small with a total of 510,340 cows in 2020, of which 303,390 for milk production (Jordbrukverket, 2020). According to Swedish legislation, antimicrobials are only available for treatment of animals on veterinary prescription. The major organic certification body in Sweden, KRAV,^1^ follows the EU regulations on organic production and even go further in some areas concerning animal welfare. A previous study in 30 conventional and 30 organic dairy farms showed no apparent difference in AMR despite the differences in AMU regulation between organically and conventionally managed herds (Sjöström et al., 2020) and not significantly different use of injectable mastitis treatment (Olmos Antillón et al., 2020). In the current study, we wanted to further examine AMR in the local farm environment within these Swedish conventional and organic dairy farms by extending the analyses to other potential sources as well as a more thorough genetic analysis of resistant *E. coli*.

## Material and Methods

### Sample Population and Collection

All samples were collected from 54 dairy farms across Sweden in 2017, the same farms as described in Sjöström et al. (2020), during the second sampling period of that study (27 organic and 27 conventional herds). A convenience sampling design was used for the original study (Sjöström et al., 2020). At each farm, up to 10 samples were collected, these samples were: five fecal swabs from healthy calves <2 months old; one swab from an indoor manure drainage site; one swab from the manure pit; one sample consisting of bird fecal droppings; one with rodent fecal droppings; and one collection of whole live flies picked from the fly tape in the barn. All calf samples were collected rectally with an Amie’s charcoal culture swab (Copan diagnostics Inc., Murrieta, CA, United States) and the manure samples were collected with E-swabs (Copan diagnostics Inc., Murrieta, CA, United States), while fecal droppings from the bird and the rodent samples were placed in tubes with Amies agar gel with charcoal transport media and whole flies were crushed in tubes with Amies agar gel with charcoal transport media. All collected swab samples were immediately placed into transport tubes with Amies agar gel with charcoal transport media as well as other samples and stored at 4°C after collection and were continued to be stored at 4°C at the laboratory facility before sample processing begun.

### Bacterial Isolation and Antimicrobial Susceptibility Testing

After collection all samples were subjected to indicator *E. coli* isolation within a week of their collection date at the Zoonosis Science Center, Uppsala University. All samples were diluted in 3 ml of 0.9% NaCl and, subsequently, 1 ml of 10-fold dilutions (10^−2^ and 10^−4^ for calf samples, 10^−1^ and 10^−3^ for other samples) were streaked on Petrifilm™ (3M™, St Paul, MN, United States) Select *E. coli* count (SEC) plates (3 M Microbiology Products) and cultured overnight at 42°C. Simultaneously, all samples were enriched in 4 ml of peptone water overnight at 37°C and subsequently streaked on cephalosporin CHROMAgar C3G selection plates (CHROMagar, Paris, France) and incubated at 37°C overnight. From the Petrifilm SEC plates one random colony was collected and subcultured on Horse Blood Agar plates and identified as *E. coli* by morphology and the indole test. From the CHROMAgar C3G plates, pink colonies were identified as *E. coli* by morphology and an indole test and were also subcultured. All isolates were antimicrobial susceptibility tested by the VetMIC™ GN-mo (version4; SVA, Uppsala, Sweden) broth microdilution microtiter panel of 13 antimicrobial substances (ampicillin, ceftazidime, ciprofloxacin, chloramphenicol, cefotaxime, florfenicol, gentamicin, kanamycin, nalidixic acid, streptomycin, sulfamethoxazole, tetracycline, and trimethoprim) performed in the laboratory facility at the Zoonosis Science Center, Uppsala University.

### DNA Extraction and Whole Genome Sequencing

All isolates in this study were re-streaked onto Luria Bertani (LB) plates and incubated at 37°C for 18 h. Following culture, a check for potential contamination was done and 3 ml overnight cultures were made with one fresh colony into LB agar broth, incubated at 37°C for 18 h. DNA was extracted from the overnight culture using the Qiagen DNeasy blood and tissue kit in accordance with the manufacturer’s instructions (Qiagen, Hilden, Germany). For all DNA extracts DNA concentration was calculated were performed using the dSDNA HS Assay kit on the Qubit Fluorometer (Thermo Fisher Scientific, Fair Lawn, NJ, United States) and OD260/280 in the range of 1.8–2.0 verified by the NanoDrop spectrophotometer (Thermo Fisher Scientific, Fair Lawn, NJ, United States). All DNA extracts were lyophilized and then shipped at room temperature to the Novogene sequencing facility (Novogene, Hong Kong, China) and sequenced on the Novoseq Illumina platform (Illumina, San Diego, CA, United States) which produced approximately 1 GB of 150 bp pair-end sequencing reads per isolate.

### Antimicrobial Resistance Profiling and Genomic Analysis

**Figure 1:**
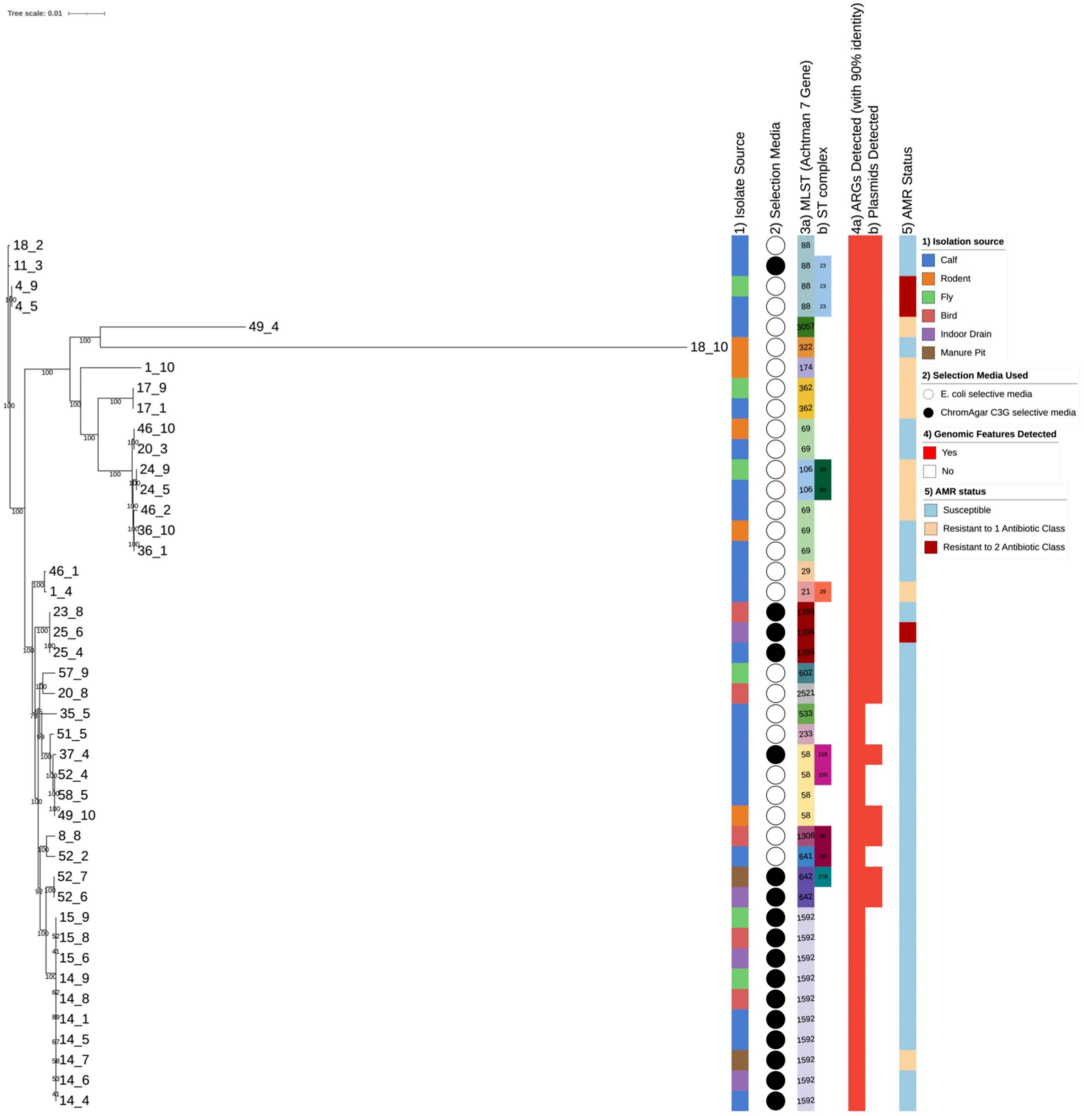
Maximum Likelihood phylogenetic tree of the bacterial isolates of this study. The tree includes all isolates sequenced named by farm of origin followed by sample source type. It was constructed using core genome of each isolate with 100 bootstrap replicates with values on the tree and the corresponding metadata of (1) isolate sample source according to key, (2) isolate selection media used according to key, (3) isolate genomic typing (3a) multi-locus sequencing typing using Achtman 7 gene scheme and (3b) detection of sequence type clonal complexes, (4) genomic features in the *E. coli* isolates (4a) detection of antimicrobial resistance genes (4b) detection of plasmid replicons according to key, (5) the *E. coli* isolates phenotypic AMR status to tested drugs according to key.

**Figure 2:**
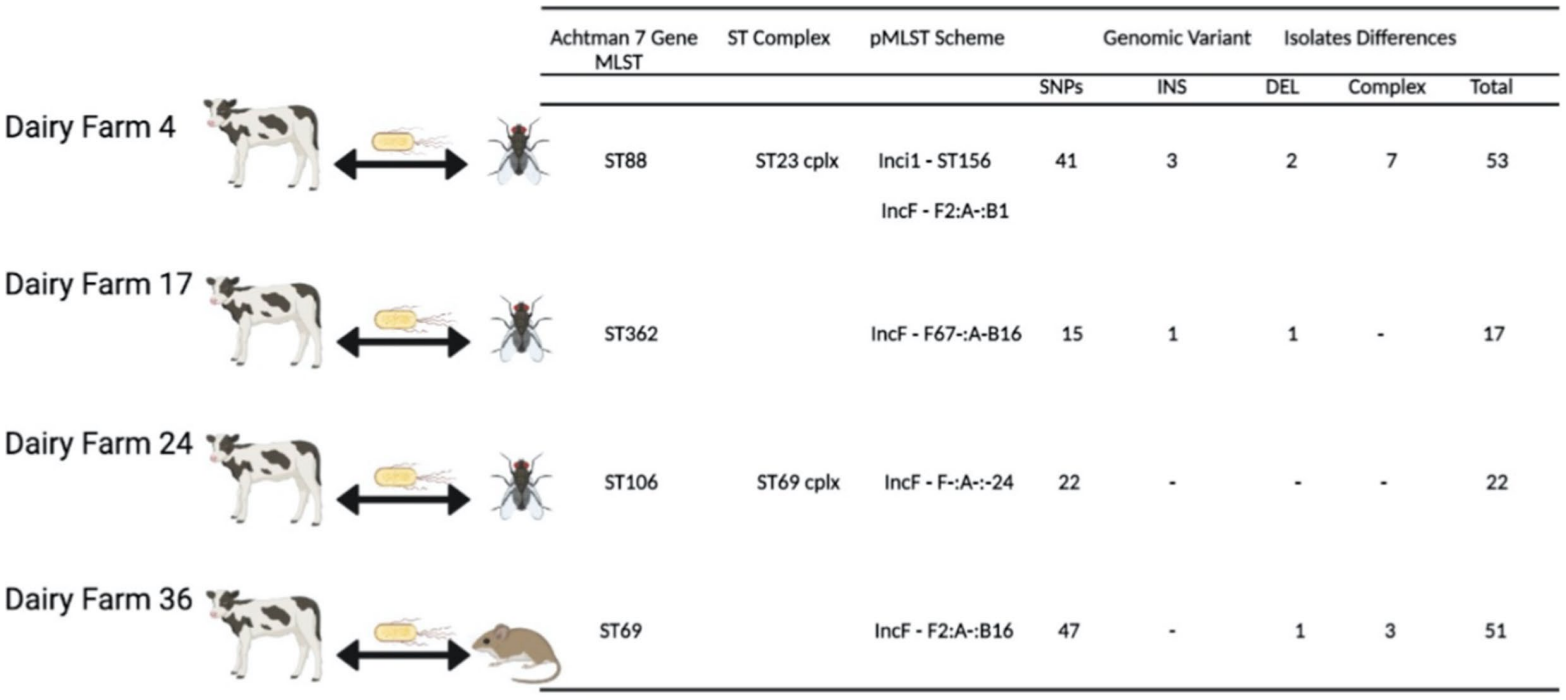
Bacterial isolates from two different sources in the same farm that share the same Achtman 7 Gene MLST, ST complex number is stated when available and pMLST types for plasmid incompatibility groups are given for IncF and Inci1 plasmids. Genomic variants differing among isolates are sorted by types (SNP = single nucleotide polymorphism, INS = genomic insertion, DEL = genomic deletion, Complex = genomic difference that could be comprised of single nucleotide polymorphism and or genomic insertion and or genomic deletion).

All antimicrobial susceptibility test (AST) data were converted into either a susceptible, intermediate or resistant classification for each isolate and antibiotic drug in accordance to EUCAST clinical breakpoints (EUCAST, 2018). The use of clinical breakpoint for human medicine was due to our interest in the potential zoonotic threat, however not all tested antibiotic drugs had a clinical breakpoint. These data were then further processed in Python (3.8.3rc1 Documentation, 2020) using the matplotlib, pandas and seaborn packages. All processing of the whole genome sequences was done with open software with an in-house bioinformatics pipeline as described in Hickman et al. (2021). The genomic data processing was done on the Uppsala Multidisciplinary Centre for Advance Computational cluster (UPPMAX). Using the described pipeline we generated a molecular report file (Supplementary File 1) to compile all detected antimicrobial resistance genes (ARGs), extra-chromosomal plasmids, sequence types by computationally multi-locus sequence typing (MLST), plasmids by plasmid multi-locus sequence typing (pMLST); and a Maximum Likelihood phylogenetic tree to assess isolate relatedness from the different farms and sample sources that was subjected to 100 times bootstraps, with confidence values included on the phylogenetic tree (Figure 1) For isolates from the same farm, where the same MLST sequence type was found and pMLST sequence type for the detected plasmids, further SNP analysis was done using Snippy version 4.0.5 (Seemann, 2015; Figure 2).

### Overview of Study Isolate Collection

Forty-three *E. coli* isolates were included in this study, comprising all *E. coli* isolates that grew in the selective culturing (on C3G plates, 17 isolates). Furthermore, of the strains isolated from the non-selective culturing (on Petrifilm), the 26 isolates demonstrating resistance to the highest number of tested antibiotics were included. This subset bacterial collection originated from 21 dairy farms where 11 farms had isolates from both a calf fecal sample and at least one environmental sample (from manure drain, manure pit, rodent feces or bird feces, and whole flies).

Isolates were compared for genomic relatedness and genomic determinants between each isolate source, when possible (1) between calves and rodent samples, (2) between calves and bird samples, (3) between calves and fly samples, (4) between bird and rodent samples, (5) between rodent and fly samples, and (6) between bird and fly samples. When possible, manure drain and manure pit samples were also compared as a reference, although there were only 4 and 2 isolates, respectively.

### Ethical Approval

This study was done in accordance with the Swedish regulations, the competent authorities stated that no ethical permission was required for the sampling and the veterinarian handled the animals according to relevant ethical standards. All participating farmers were informed of the purpose and methods of the study, that participation was voluntary and anonymous and that they had the right to withdraw from the study at any time.

### Data Availability

Raw sequence data can be obtained from the European Nucleotide Archive (ENA) under the project accession number PRJEB45447. All sequence data from computation workflow is compiled in Supplementary File 1 and interspecies genomic difference data in Supplementary File 2.

## Results

### Genomic Isolate Typing and Core Genome Analysis

Within our data we generally observed that isolates from the same farm clustered despite coming from different sources and the isolates from the same farm often had the same MLSTs (multi locus sequence types; Figure 1). This was clearly observed in farm 14 which had seven isolates sequenced: three from calves, and one each from the drain, well, bird, and fly sources, which all had Achtman 7 gene MLST ST-1592 (Figure 1—Part 3a and b). It is also important to note, that in a few cases different ST types were seen on the same farm. In the cases where different *E. coli* isolates ST were found within the same farm, we found the different ST *E. coli* isolate clustered together with those from other farms rather than with isolates from the farm where the isolate was acquired, e.g., calf isolate 46_1 from farm 46 which was ST-29 and was on a different branch of the phylogenetic tree in comparison to the other calf isolate 46_2 and the rodent sample that were both has ST-69 (Figure 1). In farms 4, 17, and 24 samples from calves and flies shared the same ST, while in farms 36 and 46 calves and rodent samples shared the same ST, and in farm 15 bird and fly samples had the same ST.

### Genomic Isolate and pMLST

The combining of MLST and pMLST results demonstrated interspecies sharing of clone types with the same mobile genetic elements. In seven farms, two or more sources shared the same MLST, when combined with the pMLST results there were four farms where both the same MLST and pMLST were detected in isolates from different sources (Figure 2). Due to the low number of nucleotide differences between the two isolates and the fact that they also harbored the same plasmids, potential clonal sharing between the two host species is highly likely.

### Phenotypic Antimicrobial Susceptibility Testing Coupled With Whole Genome Sequencing

The phenotypic susceptibility patterns in the isolates from calves and manure have been previously published (Sjöström et al., 2020). We plotted the AST results to each of the 13 drugs for each isolate according to their sample group (Supplementary Figure 1). From the group distribution plots we were unable to find correlations between groups, possibly due to the low number of isolates. Using EUCAST clinical breakpoints, we observed a generally low AMR prevalence; we did see that 21% of isolates (9/43) were resistant to one class of antibiotic drugs in the phenotypic AST data this was most frequently ampicillin or trimethoprim (Supplementary File 3). We also observed 7% of isolates (3/43) that were resistant to antibiotics from two different drug classes, the three isolates that were resistant to both ampicillin and trimethoprim (Supplementary File 3).

**Figure 3:**
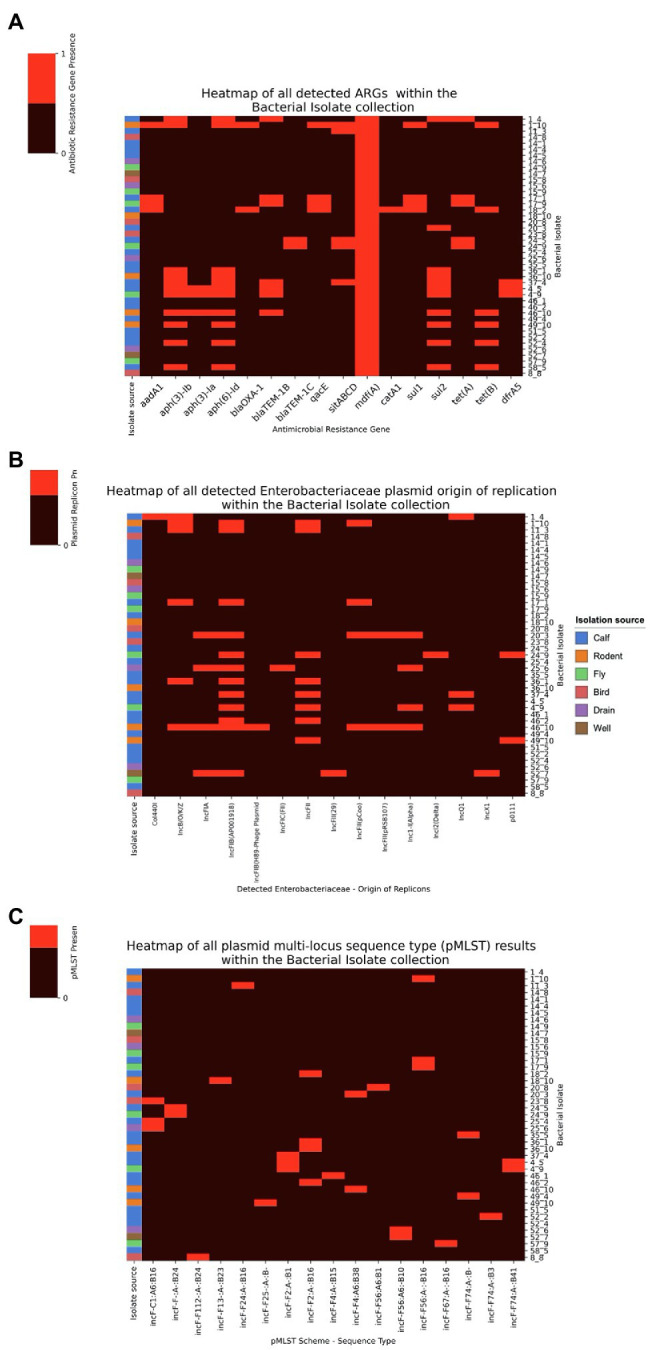
Heatmaps showing the presence of ARGs and plasmid replicons and pMLSTs discovered in bacterial isolates from Swedish dairy farms. **(A)** Heatmap of all detected ARGs in the bacterial isolates with isolation source annotated in accordance to the key. **(B)** Heatmap of all detected plasmids origin of replications in the bacterial isolates with isolation source annotated in accordance to the key. **(C)** Heatmap of pMLST in the bacterial isolates with isolation source annotated in accordance to the key.

From our WGS results we were able to detect ARGs, AMR-related chromosomal mutations, plasmid replicons, virulence factors and plasmid types. In all isolates, 1 or more ARGs were detected and in 65% of isolates, extra-chromosomal plasmids were detected (Figure 1—Part 4a and b). In all sequenced isolates regardless of source, the *mdfA* gene was present; this gene encodes for the MdfA multi-drug efflux pump (Figure 3A). In the drug resistant (ampicillin and trimethroprim) isolate 4_5 (isolate source calf), we detected a plethora of ARGs (*blaTEM-1B*, *dfrA5*, *mdfA*, *tetA* and *sitABCD*) where *blaTEM-1B* (Seenama et al., 2019) and *dfrA5* (Thungapathra et al., 2002) are known to be responsible for the conferred resistance phenotype. In the drug resistant (ampicillin and trimethroprim) isolate 25_6 (isolate source drain), the only ARG was *mdfA* but further analysis in PointFinder (Zankari et al., 2017) revealed multiple chromosomal mutations in *ampC*, *16S rrsB*, *16S rrsC*, *16S rrsH*, *23S*, *parC* and *pmrB* genes that might contribute to the resistance phenotype (Supplementary File 1). In the gentamicin resistant isolate 46_2 (isolate source calf), we detected the ARG *mdfA* and several RNA gene mutations *16S rrsC*, *16S rrsH*, *23S* that could confer gentamicin resistance *via* the mechanism of gentamicin binding to the 30S ribosome preventing protein synthesis (Eric Scholar, 2007), we also detected other chromosomal mutations in the *gyrA* and *pmrB* genes (Supplementary File 1).

In our isolate collection, we detected 15 different plasmid replicons (Figure 3B). From our pMLST results all detected plasmids belong to either the incl1 or incF plasmid incapability groups (Figure 3C) both being clinically related groups due to the ARGs and virulence factors that can be carried in Enterobacterales (Mshana et al., 2013; Rozwandowicz et al., 2018).

## Discussion

Our study focused on *E. coli* isolates within the dairy farm environment where environmental samples and animal host samples were taken. From this we were able to see the difference in molecular characteristics of isolates from different sources and different farms as well as potential interspecies clone sharing.

In regards to phenotypic AMR, as reported in our earlier publication (Sjöström et al., 2020), the prevalence of AMR—especially MDR—was exceptionally low in our study compared to data from other countries (Van Boeckel et al., 2015). The isolate collection in the present study was heavily biased for antibiotic-resistant strains as we used isolates from selective culturing for beta-lactamase producing Gram negative bacteria and the most antibiotic-resistant isolates from non-selective culturing. Although stemming from a limited number of farms and based on a convenience sample, these data support the assumption that in countries with strict AMU, such as Sweden, there is a low baseline of AMR. The most frequently administered antibiotic classes for sick dairy animals in Sweden are beta-lactams, usually in the form of penicillin G for the treatment of mastitis, or combinations of sulphonamide and trimethoprim (Olmos Antillón et al., 2020) although the latter is being phased out with new treatment recommendations. It is therefore unsurprising that this was the most frequently observed phenotypic and genotypic antimicrobial resistance in our data in regards to sulphonamide and trimethoprim resistance, we also observed high levels of ampicillin-resistant *E. coli* isolates observed within our data. Despite low baseline of AMR in our isolates from our phenotypic analysis we were able to cultivate 17 isolates on selective culture, which surprising had low to no resistance to 3rd generation cephalosporins. We suspect this may be due to modulations of copy number of ARGs within these isolates therefore demonstrating the rapid adaption to antibiotic perturbation within the dairy farm environment (Laehnemann et al., 2014).

Within our molecular results we were surprised to find the *mdfA* gene in all the sequenced isolates. We speculate the common presence of this gene is due to the resistance effect that it could produce against various chemical substances that could be present within the dairy farm environment (Edgar and Bibi, 1997; Bohn and Bouloc, 1998; Mine et al., 1998; Lewinson et al., 2003, 2004). In addition, we did observe a large variety of ARGs and AMR related chromosomal mutations despite low AMU. We speculate that this may be the results of antibiotics use periodically for treating sick animals, or a result of scavenger animals picking up ARGs from other sources (Vittecoq et al., 2016; Swift et al., 2019; Plaza-Rodríguez et al., 2021). Therefore, a part of the on-farm bacterial population will maintain these ARGs that will rise in the population when the relevant antibiotics are used. We saw a diversity in ARGs, which is likely to be a result of their dissemination by both clonal and horizontal gene transfer (HGT) mechanisms, while chromosomal mutations tend to be disseminated by clonal inheritance. In addition, within our sequenced isolates we detected extra-chromosomal plasmids showing that HGT could be occurring, with incF plasmid incapability groups being the most common. By whole genome sequencing we were able to characterize the isolates at higher resolution. However, due to the limited number of isolates from each farm that were whole genome sequenced we can see only a snap-shot of the ARGs, AMR-related chromosomal mutations and plasmids. Future studies to further explore our findings could involve collecting more independent source samples, e.g., other animal sources in the farm environments, selecting more bacterial isolates from each sample, and using direct sequencing by metagenomic methods to examine on genomic characteristics such as presence of ARGs and extra-chromosomal plasmids within the sample. With phenotypic AST the number of isolates that can be processed is limited and hence the combined phenotypic and genotypic characterization of isolates will not yield the same multitude of information.

The comparison of *E. coli* isolates from multiple sources showed that in 7 out of 11 farms (64%), there was sharing of MLSTs. The same MLSTs were found in both animal swabs or feces and manure collection drains and pits in the environment, further supporting on-farm environmental spread of fecal bacteria from the animals, which was also observed by Massé et al. between calves and manure pits (Massé et al., 2021). On four farms (4, 17, 24, and 36) we saw both MLST and pMLST type match for *E. coli* isolates from different animal host samples. By whole genome sequencing we were able to see the genomic variation between the isolates; the low genomic variation observed between some isolate pairs constitutes strong evidence for cross-species *E. coli* potential clone sharing. This occurred on three farms between calf and fly sample and on one farm between calf and a rodent sample, demonstrating that clonal sharing of *E. coli* strains with extra-chromosomal plasmids does occur within the dairy farm environment such as between calves and other farm-based scavenger animals. We were unable to specify clone sharing due to the lack of set criteria for clonality by genomic differences in *E. coli* isolates unlike other bacterial species such as *Pseudomonas aeruginosa* (Lee et al., 2003).

We found previous reports of potential clone sharing between livestock cattle and birds (Fahim et al., 2019), as well as livestock cattle and deer (Singh et al., 2015). We did not obtain any samples from humans in the vicinity, such as livestock handlers. It would have been interesting to examine any potential zoonotic clone sharing but this could be an inclusion point in future studies. By whole genome sequencing rather than PCR methods we were not only able to see MLST types but due to the higher resolution from WGS we were also able to compare and observe genomic variation on a molecular level. Due to the limited number of available isolates for MLST, pMLST and genomic variation comparison, cross-sample dissemination rates could not be estimated. Nonetheless, we clearly demonstrate its occurrence and hope to further explore this on a larger scale to obtain a better picture of clone sharing potential and even investigate potential carriage in each investigated sample source to have a better idea of how AMR transmission occurs on dairy farms with low AMU.

## Conclusion

In Sweden, a country with strictly regulated and comparatively low animal AMU, a low baseline phenotypic and genomic AMR was observed, supporting the notion that all AMU selects for AMR on dairy farms. Our results indicate on-farm transmission of *E. coli* clones between different host species within the dairy farm environment, which has implications for the risk of environmental AMR spread.

## Data Availability Statement

The datasets presented in this study can be found in online repositories. The names of the repository/repositories and accession number(s) can be found in the article/Supplementary Material.

## Author Contributions

KS, UE, NF, SS-L, and JJ designed the study. KS collected the original samples. VA, KS, and RH did the laboratory work. RH analyzed the data and prepared figures and wrote the manuscript with critical revisions by JJ. RH and VA interpreted the data. All authors contributed to the article and approved the submitted version.

## Funding

This project was supported by the Swedish Research Council Vetenskapsrådet (to JJ, grant no. 2016-02606) and by Formas—The Swedish Research Council for Environment, Agricultural Sciences, and Spatial Planning grant no. 2014-281. The genomic data processing was enabled by resources in project [SNIC 2019/8-265 and SNIC 2019/8-11] provided by the Swedish National Infrastructure for Computing (SNIC) at UPPMAX, partially funded by the Swedish Research Council through grant agreement no. 2018-05973.

## Conflict of Interest

The authors declare that the research was conducted in the absence of any commercial or financial relationships that could be construed as a potential conflict of interest.

## Publisher’s Note

All claims expressed in this article are solely those of the authors and do not necessarily represent those of their affiliated organizations, or those of the publisher, the editors and the reviewers. Any product that may be evaluated in this article, or claim that may be made by its manufacturer, is not guaranteed or endorsed by the publisher.

## Footnotes

^1^ www.krav.se
