# Supplementary material for: Dissemination of Resistant Escherichia coli Among Wild Birds, Rodents, Flies, and Calves on Dairy Farms
Source: Front Microbiol. 2022 Apr 1;13:838339. doi: 10.3389/fmicb.2022.838339 (PMC9010975; doi:10.3389/fmicb.2022.838339)
Supplement: Supplementary file 3 [file Table_3.DOCX]

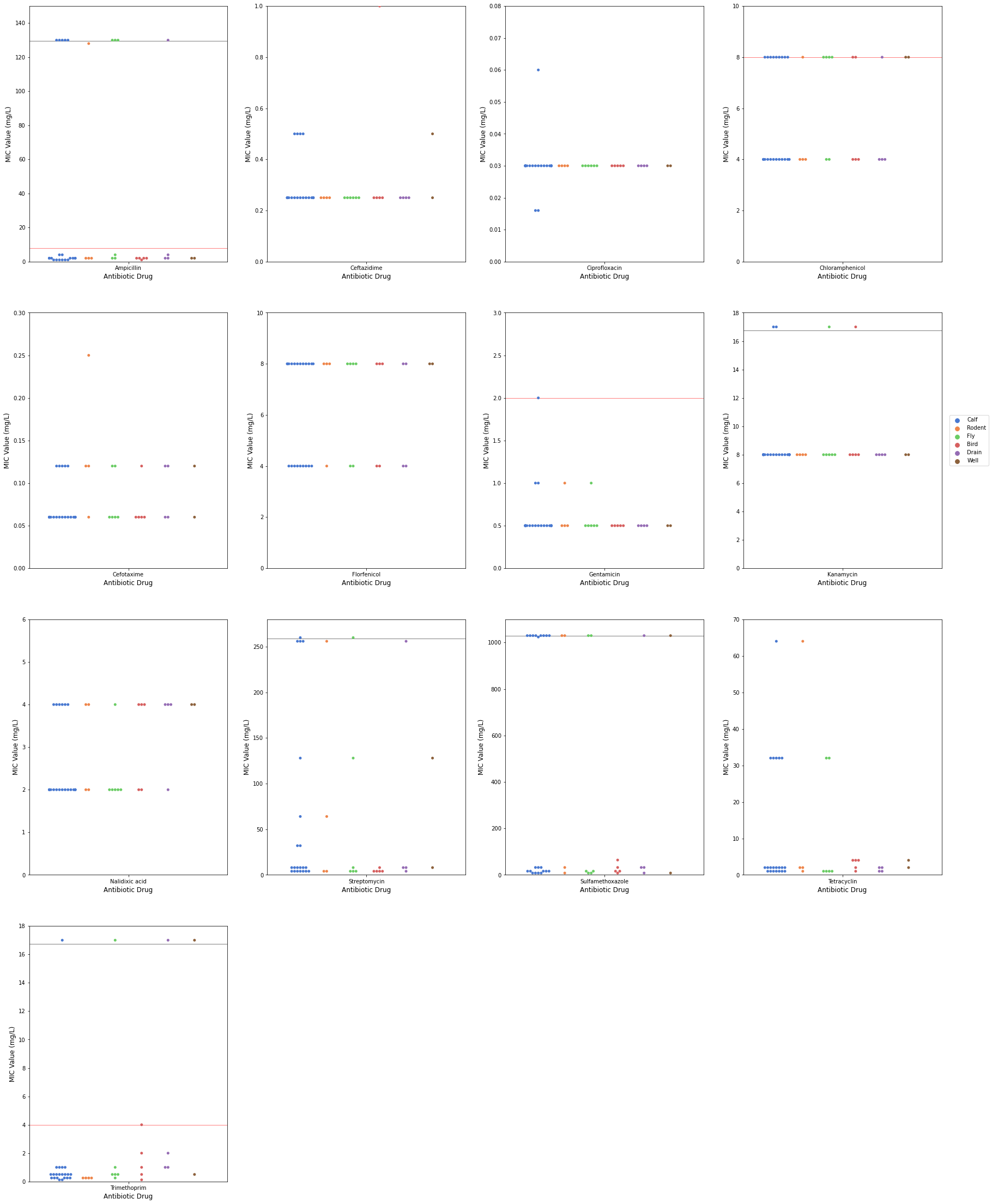


**Supplemental Figure 1. Minimum Inhibitory Concentration (MIC) distribution for each isolate within each sample source group for 13 antibiotics drugs.** VetMIC test upper limit is displayed as a line at the top of the y axis. For isolates above this line, the MIC could not be validated any further. EUCAST clinical breakpoints are displayed on the y axis for each drug as a red line.
